# Influence of Cowpea Plants on Soil Bacterial Community and Soil Quality: Effects of the Rhizosphere

**DOI:** 10.1002/pei3.70157

**Published:** 2026-05-10

**Authors:** Motlagomang Khantsi, Olubukola Oluranti Babalola

**Affiliations:** ^1^ Food Security and Safety Focus Area, Faculty of Natural and Agricultural Sciences North‐West University Mmabatho South Africa

**Keywords:** bacterial diversity, microorganisms, plant health, plant‐microbe interactions, soil fertility

## Abstract

Cowpea ( *Vigna Unguiculata* ), a vital legume for suitable agriculture and food security in sub‐Saharan Africa, plays a crucial role in improving soil health through intricate plant‐microbe interactions in the rhizosphere. This review synthesizes current knowledge on the microbial interactions in the rhizosphere, focusing on soil health, microbial diversity, and their contributions to nutrient cycling and plant growth. Cowpea roots foster a diverse microbial consortium, including nitrogen‐fixing rhizobia, phosphate‐solubilizing bacteria and organic matter decomposers, which enhance soil fertility and structure. The microbial community in the cowpea rhizosphere is shaped by complex soil physiochemical properties, such as potential of hydrogen (pH), nutrient availability, and salinity, which significantly influence plant‐microbe interactions. However, contradictions persist regarding pH's effect on microbial diversity, with unresolved questions about how specific environmental conditions regulate microbial taxa. Advanced techniques, including metagenomic analyses, have provided deeper insights into the taxonomic and functional composition of rhizosphere microbiomes, uncovering both abundant and rare microbial taxa involved in these processes. Despite these advancements, gaps remain in understanding the dynamic responses of microbial communities to environmental stresses. Bridging these gaps through integrative multi‐omics approaches will enable the development of microbiome‐informed strategies to improve cowpea productivity and promote sustainable agricultural practices, ensuring resilience in the face of climate variability.

## 1 Introduction

Food safety and security represent pressing global challenges, particularly in sub‐Saharan Africa, where agricultural yields remain considerably lower than in other developing regions (Omomowo and Babalola 2021; Odey et al. 2024). The United Nations Food and Agriculture Organization (FAO) defines food security as a condition in which all people, at all times, have physical, social, and economic access to sufficient, safe, and nutritious food that meets their dietary needs for an active and healthy life. Rapid global population growth combined with severe climate change is exacerbating pressures on agricultural systems, causing land degradation, altered soil properties, and disrupting essential processes such as nutrient cycling and plant–soil interactions (Abobatta et al. 2021). Addressing food security in Africa and globally demands urgent, market‐driven transformations across the entire food system, not merely agricultural production (Onwujekwe and Ezemba 2021).

Cowpea ( *Vigna unguiculata* L. Walp) is a nutritionally rich and highly adaptable legume that plays a crucial role in sustainable agriculture, particularly in semi‐arid and tropical regions (Nounagnon et al. 2024; Kim et al. 2024). In South Africa, it ranks as the third most important grain legume, after groundnuts and soybeans, with significant production in provinces such as Limpopo, Mpumalanga, and KwaZulu‐Natal (Zondi et al. 2022). Globally, cowpea cultivation extends across West Africa, parts of Asia, and the Americas, supporting both subsistence and commercial farming systems (Singh 2020; Badiane et al. 2014; Silva et al. 2018). The crop's low water requirements and resilience to drought make it especially valuable for marginal environments. Beyond its role as a food source, which is provides high protein content and serves as a meat substitute, cowpea is also essential as fodder and for income generation among smallholder farmers (Ahmed et al. 2023; Crizel et al. 2023; Popoola et al. 2022; Omomowo and Babalola 2021).

**FIGURE 1:**
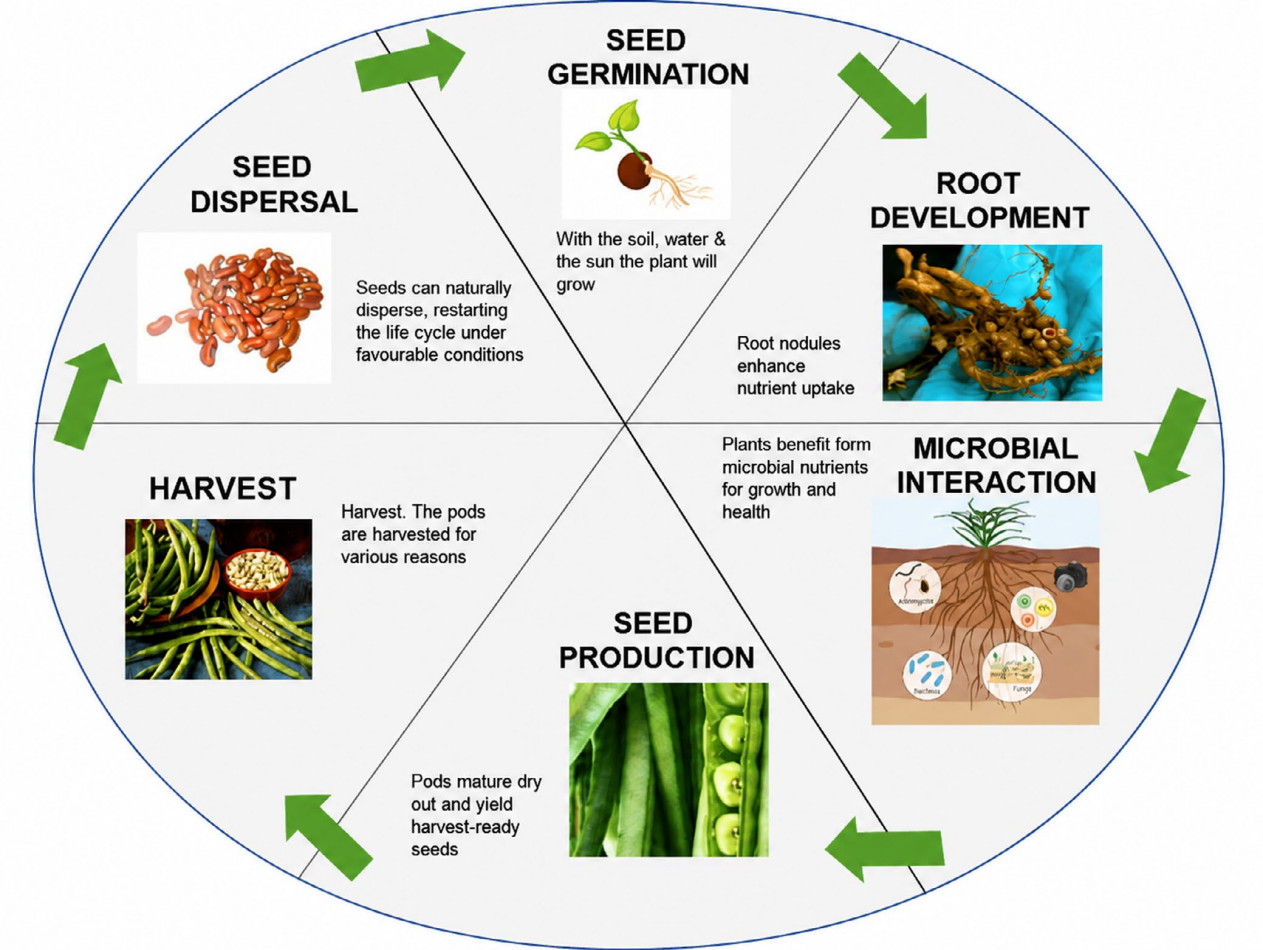
The developmental stages of the cowpea plant.

Cowpea contributes to improving soil health through its root system and rhizosphere activities. It exudes a complex mixture of organic compounds (e.g., sugars, amino acids, vitamins) into the soil, which selectively enriches specific microbial communities (de Araujo et al. 2019; Chen and Liu 2024). The rhizosphere harbors beneficial plant‐growth‐promoting rhizobacteria (PGPR), which enhance nutrient acquisition, suppress soil‐borne pathogens, and bolster plant immune responses (Agbodjato and Babalola 2024). Throughout the cowpea cycle, microbial communities actively support plant health and productivity. Microbial roles such as nitrogen fixation, nutrient mobilization, disease resistance, and stress tolerance are integral to the plant's growth, from seed germination to harvest (Figure 1). These interactions between plant and these beneficial microbes ensure improved nutrient use efficiency, enhanced plant health and sustainable soil fertility. This synergy between cowpea and its rhizosphere microbiota highlights the importance of microbial ecosystems in promoting sustainable agriculture and enhancing crop resilience under variable environmental conditions (Mahmud et al. 2020; Leite et al. 2016).

Despite the recognized importance of cowpea‐microbe interactions, several critical knowledge gaps remain. There is limited understanding of how soil physicochemical properties and microbial community dynamics interact under varying environmental stresses, such as drought and salinity, especially in different agroecological zones. Data scarcity on spatial and temporal variability in rhizosphere microbial populations constrains the development of tailored bioinoculants and soil management strategies. Furthermore, mechanistic insights into the molecular signaling pathways governing cowpea‐rhizobia symbiosis and the broader microbiome assembly are still incomplete. The insufficiency of integrated studies combining soil chemistry, microbiome genomics, and cowpea genotype effects hinders the optimization of microbial‐mediated soil fertility and resilience.

This review aims to comprehensively synthesize current knowledge on the interactions between cowpea and soil microbiomes, with an emphasis on soil health and microbial ecology under varying physicochemical and environmental conditions. By identifying contradictions, knowledge gaps, and emerging research needs, it seeks to inform sustainable agricultural strategies that leverage cowpea‐microbe partnerships to enhance food security, soil fertility, and crop resilience in sub‐Saharan Africa and beyond.

## 2 Soil Properties That Influence Interactions of the Cowpea‐Microbiome

Research suggests that the soil serves as the seed bank of the rhizosphere microbiome (Ling et al. 2022). The soil microbiome underpins cowpea growth by serving as a reservoir of rhizobia and other beneficial microbes crucial for nitrogen fixation and nutrient cycling (Kumar et al. 2025). It sets the foundation for understanding the role of soil in crop productivity. Plant growth, development, and yield are intricately interrelated with the plant's microbiome, which includes rhizobia and other beneficial microorganisms.

Studies by Bhatti et al. (2026), Bhardwaj et al. (2024), and Rajput and Husain (2023) emphasize how soil chemical properties such as potential of hydrogen (pH) and salinity govern microbial diversity and functionality. Goodall et al. (2025) highlighted the direct influence of pH on agricultural systems and microbial genomic traits. Cowpea prefers neutral to slightly acidic soils (pH 6.0–8.0), supporting *Rhizobium* and *Bradyrhizobium* species that form beneficial symbiosis with the plant (Sandhu and Chaturvedi 2025). In contrast, soil acidity can reduce microbial diversity and nodulation, negatively impacting pod yield (Priya 2021). However, acid‐tolerant strains like *Pseudomonas*, *Streptomyces*, and *Bacillus* can still thrive, suggesting microbial adaptations or taxonomic differences (Naz et al. 2022; Martins da Costa et al. 2021). The contradiction necessitates further research to delineate pH thresholds for specific microbial communities.

Salinity reduces microbial diversity and nitrogen fixation efficiency (Mukhtar et al. 2020; Kirova and Kocheva 2021). While halotolerant rhizobacteria such as., *Pseudomonas* and *Bacillus* can mitigate salinity stress, they cannot fully offset its detrimental effects on cowpea cultivation (Jayawardhane et al. 2022; Zörb et al. 2019).

Nutrient deficiencies, such as nitrogen and phosphorus, limit proliferation and symbiosis with microorganisms (Waseem et al. 2023). *Bradyrhizobium* species increase nodulation efforts under nitrogen stress, but severe deficiency impairs nitrogen fixation (Mekonnen et al. 2023).

In free‐living nitrogen‐fixation bacteria like *Azotobacter* and *Azospirillum*, populations fluctuate based on nitrogen availability (Jehani et al. 2023; Aasfar et al. 2021). Phosphorus deficiency triggers organic acid exudation from cowpea roots, which influences microbial populations such as *Bacillus* and *Pseudomonas* (Soares et al. 2023). These microbes solubilize phosphorus, supporting plant growth. Phosphorus stress also enhances arbuscular mycorrhizal fungi (AFM) formation, which impacts microbiome composition (Zhao et al. 2024).

Micronutrient deficiencies, including iron (Fe), copper (Cu), and zinc (Zn), impair nutrient cycling and plant stress tolerance (Singhal et al. 2023). These essential elements are required in small amounts but play a pivotal role in cowpea growth and yield.

Key physicochemical parameters such as soil texture, soil organic matter (SOM) mineral composition, and enzyme activities remain underexplored but are critical for understanding cowpea‐microbe interactions (Hartmann and Six 2023; Ogwu et al. 2024). Soil texture influences microbial habitats, with loamy soils providing optimal conditions for microbial communities due to their balance in water retention and aeration (Hasan et al. 2024). However, there is sufficient evidence on how mineralogical variability affects the microbiome in the cowpea rhizosphere specifically.

SOM provides essential plant nutrients and supports nitrogen‐fixing microbes like Rhizobium and phosphate‐solubilizing bacteria (*Pseudomonas* and *Bacillus*), promoting cowpea growth and nodulation (Bhattacharya et al. 2024). Maintaining healthy SOM creates a more balanced soil environment, reducing pathogen incidence and improving microbial activity (Thakur et al. 2022).

Mineral composition including Calcium (Ca), Magnesium (Mg), and Phosphorus (P) is crucial for microbial interaction in the rhizosphere, shaping nutrient cycling and symbiotic effectiveness (Dhaliwal et al. 2024). Studies by Yürürdurmaz (2022), indicate that cowpea rhizosphere typically exhibits higher Ca and Mg levels compared to bulk soil, supporting microbial functions necessary for plant nutrition.

Cation exchange capacity (CEC) is an important parameter, influencing nutrient availability and microbial survival (Sidsi et al. 2025), Soils with higher CEC retain more essential nutrients, promoting microbial stability and enhancing cowpea growth (Prajapati et al. 2025). In field trials, cowpea cultivation was shown to increase soil CEC, improving soil fertility (Adekiya et al. 2024).

Enzyme activities in the rhizosphere, such as acid phosphatase and alkaline phosphatase, play a crucial role in phosphorus nutrition and organic matter decomposition (Jaiswal et al. 2021). Microbial genera like *Lysobacter* and *Flavobacterium* produce enzymes that enhance phosphorus solubilization and promote plant growth, with microbial composition influencing enzyme activity (Solangi et al. 2024).

Climate factors, such as temperature and moisture, also significantly affect microbial activity and plant‐microbe interactions. Favorable climates enhance rhizobacterial activity, improving cowpea growth through nitrogen fixation and nutrient solubilization (Ayalew and Yoseph 2022).

Topography influences cowpea microbiome interactions by affecting water drainage and microbial habitats (Liu et al. 2020). While more research is needed on slope‐related microbial interactions, *Pseudomonas fluorescens* and *Bacillus subtilis* are known for their resilience and ability to stabilize soil on sloped terrains as studied by Solanki et al. (2020).

There is a gap in linking specific microbial taxa to biogeochemical functions, due to functional redundancy and shifting microbial activity. To address this, research using advanced techniques like shotgun metagenomics is essential for understanding soil health, fertility, and resilience (Akinola et al. 2021a; Lema et al. 2023).

## 3 The Cowpea Rhizosphere: A Nexus of Plant‐Microbe Interactions and Sustainable Agriculture

The cowpea rhizosphere, defined as the narrow soil zone influenced by root exudates (Choudhary et al. 2022) serves as a key interface for complex plant–soil–microbe interaction. It hosts a rich diversity of microorganisms, including bacteria, fungi, archaea, and viruses (Akinola et al. 2021b). Prominent bacterial groups such as Actinobacteria, *Bacillus*, and *Pseudomonas* are essential competitors contributing to various functions (Lata et al. 2025). These diverse microbial communities provide important ecosystem services, promoting plants' growth and soil quality that are fundamental for sustainable agriculture (Omomowo and Babalola 2021).

The cowpea rhizosphere harbors stress‐tolerant bacteria such as *Priestia filamentosa* and *Bacillus aryabhattai* that thrive under drought and nutrient‐limited conditions (Abiala et al. 2023). Other bacteria, including *Azotobacter* and *Bacillus* species, produce plant hormones and protective metabolites that enhance resilience to abiotic stresses like drought and salinity (Gamalero and Glick 2022). These microbes contribute functional redundancy, stabilizing the rhizosphere ecosystem under environmental fluctuations and improving cowpea stress tolerance.

Beneficial rhizosphere microorganisms supress soil‐borne pathogens by producing antibiotics such as circulin 2, 4‐diacetylphloroglucinol (DAPG) (Agbodjato and Babalola 2024; Kankariya et al. 2025; Rizvi et al. 2022). Species like *Bacillus* and *Pseudomonas* inhibit pathogens such as Fusarium fungi and induce systemic resistance in cowpea, enhancing its defense mechanisms (Ghoniem et al. 2024). Such as microbial activities, reduce dependency on chemical pesticides, supporting environmentally sustainable cowpea cultivation.

Nitrogen fixation in the cowpea rhizosphere is primarily facilitated by Rhizobia (*Bradyrhizobium* species) forming nodules that convert atmospheric nitrogen (N2) into free‐living available ammonia (NH3), via nitrogenase enzyme activity (Gorgia and Tsikou 2025). Free‐living nitrogen‐fixers such as *Azospirillum*, *Azotobacter*, *Cyanobacteria*, and *Clostridium* also contribute to nitrogen availability (Kozieł 2024; Jehani et al. 2023; Grzyb et al. 2021). Phosphate solubilizing bacteria including *Agrobacterium*, *Pseudomonas*, *Burkholderia*, and *Bacillus* produce organic acids and phosphatases to increase phosphorus availability (Mathew et al. 2024; Kaur et al. 2024). Additionally, siderophore‐producing microbes such as *Pseudomonas* enhance iron uptake, a crucial micronutrient for chlorophyll synthesis and enzyme function (Lata et al. 2025; Mishra et al. 2025; Roriz et al. 2020).

**FIGURE 2:**
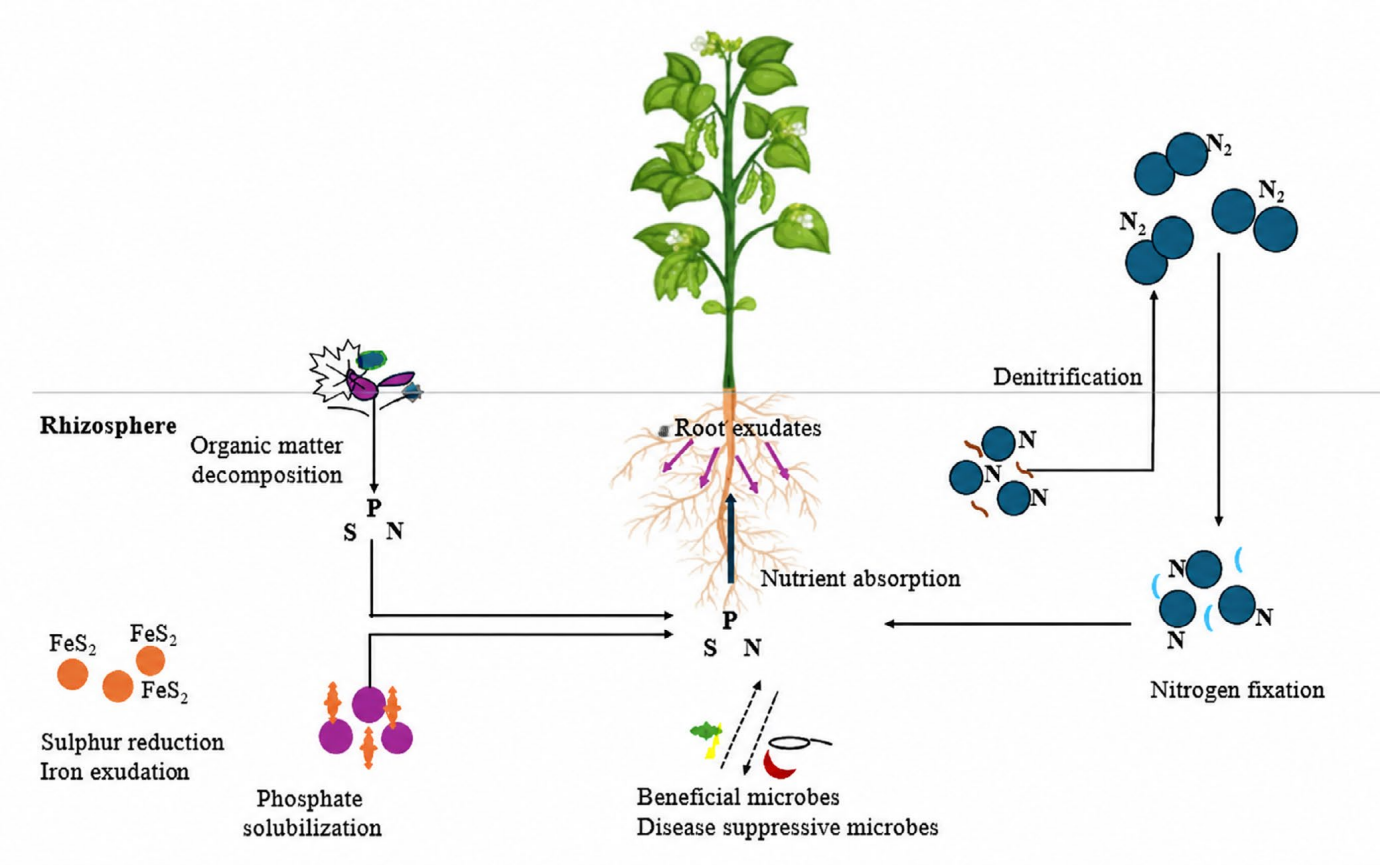
A nexus of cowpea plant‐microbe interaction.

Figure 2 illustrates this reciprocal network, showing that cowpea roots do not merely host microbes but actively select, enrich and regulate them through chemical signaling. Plant recruit microorganisms from the surrounding soil through carbon‐rich exudates, while the recruited microbes, in turn, influence nutrient acquisition, stress tolerance and disease suppression through direct and indirect interactions.

Organic matter decomposition in the cowpea rhizosphere is driven by microbial groups including *Proteobacteria*, which primarily colonize nutrient‐rich environments and are essential in the carbon cycle (Ayangbenro et al. 2022). Additional important decomposers include *Actinobacteria*, *Bacteroidetes*, and *Firmicutes*, which contribute significantly to organic mineralization and cycling of carbon and nitrogen in soil (Rocha et al. 2020; Devi et al. 2022). These processes are vital for maintaining soil fertility and nutrient availability.

The cowpea rhizosphere operates as a vibrant nexus of plant‐microbe interactions that underpin sustainable agricultural systems. By co‐ordinating microbial groups with roles in nitrogen fixation, nutrient solubilization, pathogen suppression and organic matter decomposition, it promotes soil and plant health while enhancing resilience to environmental stresses. Harnessing and managing these microbial interactions can improve cowpea productivity, maintain soil quality and fortify agroecosystems against climate variations, reducing reliance on chemical inputs and supporting environmentally friendly farming.

## 4 Deciphering Cowpea Rhizosphere Communication: Inter‐ and Intra‐Species Signaling in Microbe‐Plant Interactions Via Quorum Sensing and Symbiotic Molecules

The interaction of cowpea rhizosphere is well governed by an intricate network of chemical signaling (Chukwuneme and Babalola 2025). This interplay of biochemical signals is vital for plant‐microbe symbiosis, which influences key agricultural processes such as plant growth, nutrient acquisition, and disease suppression (Sharma et al. 2020). Among other mechanisms that facilitate these processes, quorum sensing (QS), which regulates microbial behaviors based on population density and the production of symbiotic molecules, has materialized as a central process that drives both the inter‐ and intra‐species chemical conversations (Tuan et al. 2024).

QS is a mechanism through which bacteria utilize QSMs to communicate by producing and sensing signal molecules that are diffusible, commonly known as quorum sensing molecules (QSMs). In the cowpea rhizosphere, compounds like N‐acyl homoserine lactones (AHLs), which are produced by Gram‐negative bacteria, serve as key QSMs for intra‐species communication, especially in rhizobia. These signaling molecules are responsible for enzyme secretion and biofilm formation (Shahni et al. 2025).

Inter‐species signaling occurs among different microbial species. Some microbes like *Rhodococcus erythropolis* produce enzymes that degrade or interfere with QSMs of other microbial species, which could be competitors, thus disrupting communication (Kumar 2025). According to Baker et al. (2024), sharing or competing for metabolites (phytohormones and vitamins) can shape microbial community and function. Communication through intra‐species signaling occurs within the same species. Signaling responds to environmental changes and nutrient availability. *Bradyrhizobium* species use QSMs to regulate nodule gene expression. *Pseudomonas* species enable coordination of biofilm formation and antibiotic production ( *Pseudomonas aeruginosa* ). Moreover, *Bacillus* species in QSMs regulate extracellular enzyme synthesis, lipopeptide, and plant growth‐promoting traits (Hassen et al. 2025; Vinaykumar et al. 2019; Abiala and Sahoo 2022).

Cowpea rhizosphere communication also occurs through symbiotic molecules. Plants release a complex mix of compounds from their roots into the surrounding soil, collectively known as root exudates (Rai et al. 2025). These exudates contain sugars, amino acids, organic acids, and secondary metabolites. Certain molecules within the exudates, such as flavonoids and lipo‐chitooligosaccharides (LCOs), commonly called Nod Factors, act as specific signals that attract and promote the growth of beneficial microbes. They are specialized signal molecules produced primarily by rhizobia bacteria. They trigger nodule formation, leading to nitrogen fixation through symbiotic bacteria like *Bradyrhizobium* (Prajapati et al. 2024).

**FIGURE 3:**
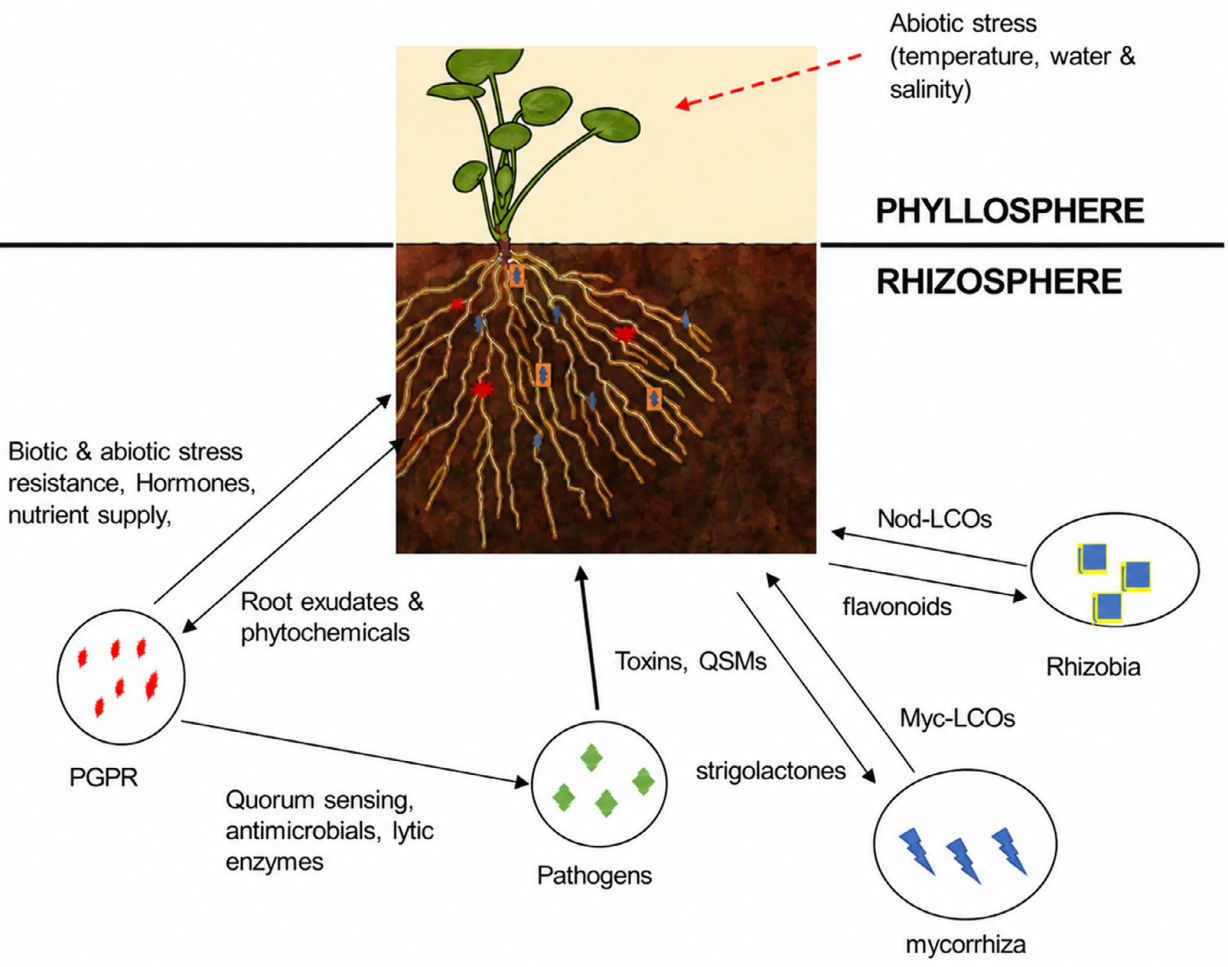
Overview of rhizosphere communication in intra‐ or inter‐species signaling among microorganisms and between microbes and plants.

In Plant‐microbe signaling, Mycorrhizal fungi form symbiosis with roots, enhancing phosphorus and nitrogen uptake. Research has shown that signals, such as Strigolactones from root exudates, attract and facilitate the attachment of beneficial fungi, often involving Myc‐LCOs, a subset of LCOs that facilitate arbuscular mycorrhizal (AM) to deal with abiotic and biotic stresses, leading to a healthier and more resilient plant (Yadav et al. 2025). Cowpea roots possess receptors to distinguish beneficial from pathogenic microbes, balancing immune tolerance and protection (Noor 2023). Microorganisms produce signals (phytohormones, LCOs/Nod, Myc) that can change plant immune responses due to various stresses, as expressed in Figure 3.

Overall, Plant‐Microbe signaling in the cowpea rhizosphere acts as a vital mediator of below‐ground communication, balancing plant growth regulation, organizing complex microbial partnerships that enhance soil fertility and plant health, thereby contributing to sustainable agricultural productivity.

## 5 Metagenomics Insights in Cowpea Plant Production

**FIGURE 4:**
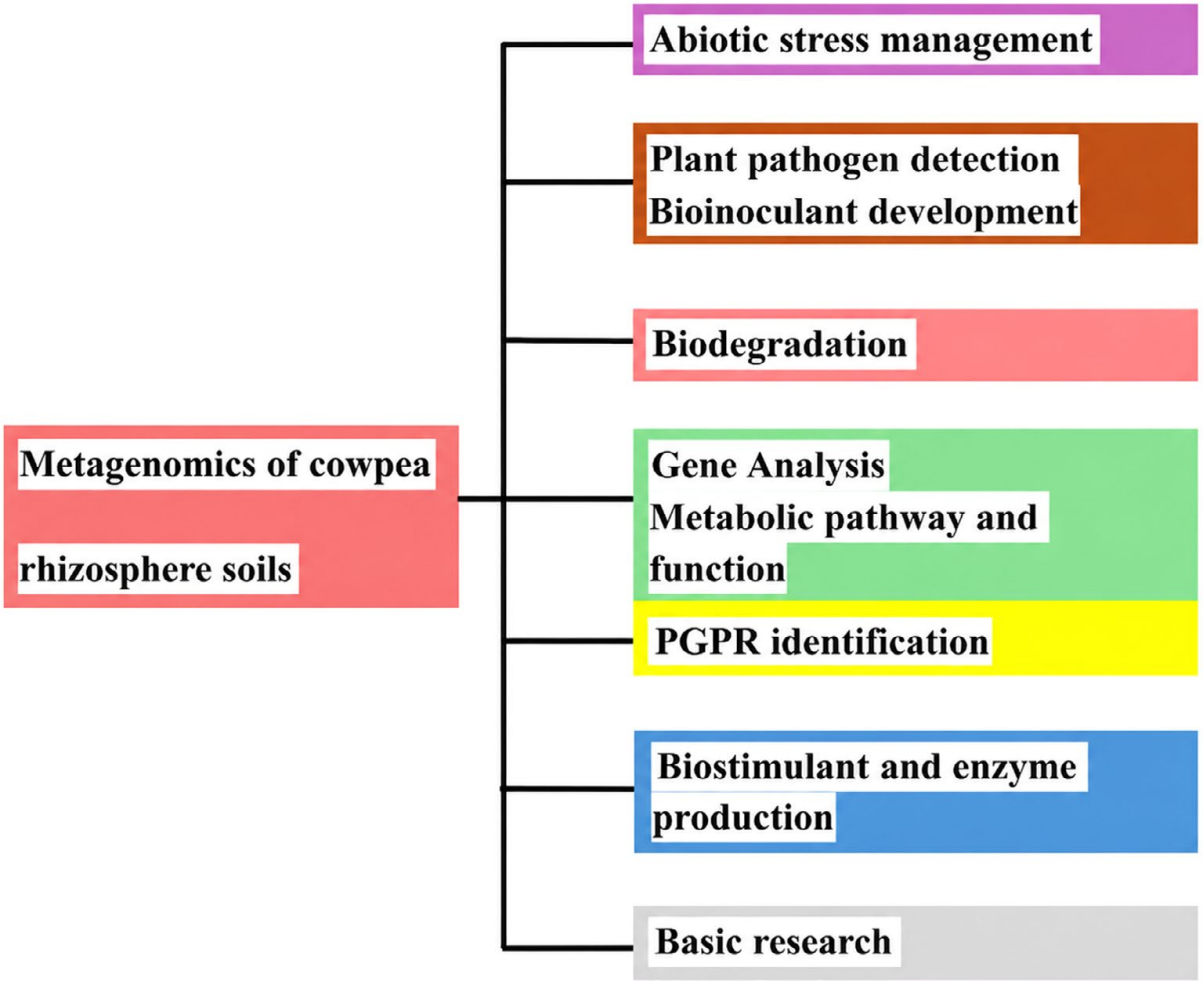
Potential applications of metagenomics in cowpea rhizosphere soils.

Metagenomics has transformed our understanding of the cowpea rhizosphere microbiome, directly influencing insights and innovations in cowpea plant production, thereby providing in‐depth insights that are shaping crop productivity and sustainable management strategies (Sarma 2024). The metagenomics approach enables comprehensive profiling of all DNA within rhizosphere soils, uncovering not only known but also previously undetected or unculturable microorganisms (Chukwuneme and Babalola 2025). Metagenomics studies have been used in various fields such as studying abiotic stresses, plant pathogen detection, facilitating bioinoculant development, and much more (Nwachukwu and Babalola 2022; Ravinath et al. 2024; Adeleke et al. 2022). Figure 4 illustrates the potential applications of metagenomics in rhizosphere soils. Microbes associated with plants or crops, including those residing in the cowpea soil rhizosphere microbiomes, play a key role in various functions essential for plant productivity. These functions encompass nutrient cycling, mineralization of SOM, and response to abiotic stresses such as temperature extremes, drought, and salinity. Specific soil microorganisms have been extensively studied for their ability to influence abiotic stress tolerance in plants. For instance, Becker et al. (2024) have observed that the rhizosphere microbiome impacts the survival of certain plant species under extreme stress conditions. Metagenomic soil surveys detect and track pathogenic viruses and fungi, enabling early warning and management strategies for emerging cowpea diseases. This broadens the horizon for integrated pest management and reduces reliance on chemical pesticides.

Metagenomics approach allows the direct quantification of functional gene abundance—such as nitrogenase genes (e.g., nifH for N fixation), phosphatases, and enzymes for organic matter turnover. Monitoring these functional markers provides actionable early indicators of soil fertility or degradation, complementing conventional chemical soil tests and enabling precision interventions in agricultural management.

By identifying key plant‐beneficial and antagonistic microbial taxa and their functional genes, metagenomics supports the creation of targeted bioinoculants (biological amendments) for cowpea production. In addition, it reveals that different cowpea genotypes can selectively recruit and enrich microbial communities (Table 1)– a foundational principle for microbiome‐informed breeding programs aimed at developing cowpea varieties with enhanced nutrient acquisition and stress resilience.

**TABLE 1:** High‐throughput sequencing (HTS) analysis in relation to cowpea plant‐microbe interaction.

Microorganism	Role in cowpea interaction	References
*Bradyrhizobium* sp.	Primary nitrogen‐fixing symbionts; form nodules on cowpea roots	Chaddad et al. (2024), Correa et al. (2024)
*Rhizobium* sp.	These nitrogen‐fixing bacteria in root nodules, relation, enhancing biological nitrogen fixation and improving soil fertility	Abd‐Alla et al. (2023), Babalola et al. (2017)
*Mesorhizobium*	Occasionally associated with legume nodulation	Alemneh et al. (2021), Naz et al. (2022)
*Bacillus* sp.	Non‐rhizobia, biocontrol agents. Produce enzymes, antibiotics and induce systematic resistance promote plant growth	Etesami (2022)
*Pseudomonas* sp.	Known for disease suppression. Moreover, known for promoting plant growth through mechanisms such as producing siderophores, antibiotics, and phytohormones, as well as biocontrol against pathogens.	Agbodjato and Babalola (2024), Ghadamgahi et al. (2022)
Actinobacteria (e.g., *Streptomyces* sp.)	Involved in organic matter decomposition and producing bioactive compounds i.e., antibiotics, that can suppress soil‐borne diseases	Gopalakrishnan and Srinivas (2019), Jacob and Sudini (2016)
*Sphingomonas* sp.	Associated with plant roots and involved in degradation of pollutants, potentially aiding plant stress tolerance	Saharan et al. (2023), Anand et al. (2023)

Metagenomics has shown that multiple microbial taxa can perform similar functions, complicating predictions of how shifts in microbial composition impact rhizosphere processes. Moreover, many findings are established in controlled environments, with limited data on long‐term effects under diverse, real world agricultural conditions; therefore, bridging current knowledge gaps with integrated “multi‐omics” studies and long‐term field research is imperative for translating these molecular insights into actionable, practical solutions for farmers.

## 6 Conclusions

The rich microbial diversity within the cowpea rhizosphere plays a significant role in enhancing soil quality by driving key functions such as nitrogen fixation, nutrient cycling, pathogen suppression, and organic matter decomposition. These microbial processes collectively improve soil fertility, structure, and water retention, which are necessary for optimal crop growth. Acting as a dynamic hub of plant‐microbe interactions, the cowpea rhizosphere supports resilient agroecosystems that mitigate environmental stresses and reduce reliance on synthetic inputs. Deciphering the dynamic inter‐ and intra‐species signaling mechanisms, such as quorum sensing and symbiotic molecules, provides a deeper understanding of microbial community regulation and plant symbioses. Metagenomic approaches have revolutionized knowledge by revealing the taxonomic and functional breadth of microbial communities, guiding the development of precision agriculture tools, targeted bioinoculants, and sustainable soil management practices. Future research integrating metagenomics with ecological and functional analyses is all‐important to elucidate in situ microbial signaling and activity, ultimately enabling the full exploitation of microbial potentials for enhancing cowpea productivity, food security, and agroecosystem sustainability under changing environmental conditions.

## Funding

The authors have nothing to report.

## Conflicts of Interest

The authors declare no conflicts of interest.

## Data Availability

Data sharing not applicable to this article as no datasets were generated or analyzed during the current study.
